# Adapting a Markov Monte Carlo simulation model for forecasting the number of Coronary Artery Revascularisation Procedures in an era of rapidly changing technology and policy

**DOI:** 10.1186/1472-6947-8-27

**Published:** 2008-06-25

**Authors:** Haider R Mannan, Matthew Knuiman, Michael Hobbs

**Affiliations:** 1 Department of Epidemiology & Preventive Medicine, School of Public Health & Preventive Medicine, Monash University, The Alfred, Melbourne, Victoria 3004, Australia; 2 School of Population Health, University of Western Australia, Perth, WA, Australia

## Abstract

**Background:**

Treatments for coronary heart disease (CHD) have evolved rapidly over the last 15 years with considerable change in the number and effectiveness of both medical and surgical treatments. This period has seen the rapid development and uptake of statin drugs and coronary artery revascularization procedures (CARPs) that include Coronary Artery Bypass Graft procedures (CABGs) and Percutaneous Coronary Interventions (PCIs). It is difficult in an era of such rapid change to accurately forecast requirements for treatment services such as CARPs. In a previous paper we have described and outlined the use of a Markov Monte Carlo simulation model for analyzing and predicting the requirements for CARPs for the population of Western Australia (Mannan et al, 2007). In this paper, we expand on the use of this model for forecasting CARPs in Western Australia with a focus on the lack of adequate performance of the (standard) model for forecasting CARPs in a period during the mid 1990s when there were considerable changes to CARP technology and implementation policy and an exploration and demonstration of how the standard model may be adapted to achieve better performance.

**Methods:**

Selected key CARP event model probabilities are modified based on information relating to changes in the effectiveness of CARPs from clinical trial evidence and an awareness of trends in policy and practice of CARPs. These modified model probabilities and the ones obtained by standard methods are used as inputs in our Markov simulation model.

**Results:**

The projected numbers of CARPs in the population of Western Australia over 1995–99 only improve marginally when modifications to model probabilities are made to incorporate an increase in effectiveness of PCI procedures. However, the projected numbers improve substantially when, in addition, further modifications are incorporated that relate to the increased probability of a PCI procedure and the reduced probability of a CABG procedure stemming from changed CARP preference following the introduction of PCI operations involving stents.

**Conclusion:**

There is often knowledge and sometimes quantitative evidence of the expected impacts of changes in surgical practice and procedure effectiveness and these may be used to improve forecasts of future requirements for CARPs in a population.

## Background

Coronary heart disease (CHD) still remains the largest single cause of death and a major cause of morbidity in most developed countries [1]. Although rates of myocardial infarction have fallen in most developed countries, hospital admission rates for CHD have continued to rise [2,3]. The treatment of CHD has meanwhile evolved, first with the widespread uptake of effective short and long-term medical treatment [4] and then with greater use of coronary artery revascularisation procedures (CARPs) [3,4] which started with coronary artery bypass grafting (CABG) during the 1970s and evolved with percutaneous coronary interventions (PCI) with the introduction and rapid use of percutaneous transluminal coronary angioplasty in the early 1980s, coronary artery stents in 1995 and more recently drug eluting stents [5]. The use of stents has contributed to a decline in the risk of a repeat CARP following PCI [5] while drug-eluting stents may lead to further reductions in these risks [6,7]. Based on several randomized controlled trials, CARPs are more effective than medical treatment in reducing mortality and morbidity from CHD [8,9].

There is great variation in rates of CARPs within and between populations with comparable rates of CHD [10,11]. Because of this uncertainty it is important to develop probabilistic methods that model the requirements for CARPs at the population level by capturing movement of individuals from one CHD/CARP state to another based on the epidemiology and natural history of CHD. This can be modelled by using Markov Monte Carlo simulation.

This study is facilitated by the availability of a unique Western Australian health information system which allows identification and linkage of hospital admission and deaths records pertaining to single individuals, thereby providing a complete event history for CHD patients [12]. In a previous paper we have described and outlined the use of a Markov Monte Carlo simulation model for predicting CHD incidence and requirements for CARPs for the Western Australian population [13]. In this paper, we expand on the use of this model for forecasting CARPs in Western Australia with a focus on how the standard model may be adapted to achieve better forecasting performance if anticipated changes are incorporated. The 1995 Western Australian population cohort is used to illustrate this approach with comparison of the predicted and actual numbers of CARPs performed over the period 1995 to 1999.

## Methods

Statewide datasets comprising (linked) cardiovascular hospital admissions for all persons who had a cardiovascular-related admission for the period 1980 to 2001 together with all deaths and population census information were used to establish CHD/CARP event history for population cohorts and estimate (population) probabilities of certain CHD and CARP events and deaths [13].

**Figure 1:**
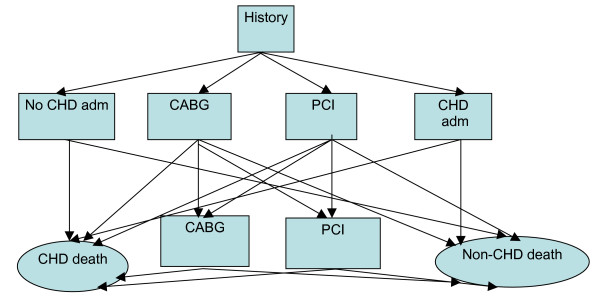
Schematic diagram for the recognised events during a cycle (year).

Under the Markov Monte Carlo simulation approach, for the cohort comprising the population of Western Australia aged 35–79 years at the beginning of 1995, model simulations are conducted to predict the number of CARPs (and other CHD events) that occur within the cohort over the next 5 years. Under our Markov model the cohort is split into initial history state groups (using their known CHD/CARP history from the linked health information system data back to 1980). The history states are (1) a history of having a PCI some time in the past; (2) a history of having a CABG some time in the past; (3) a history of having a CHD admission but no CABG or PCI some time in the past; and (4) no history of CHD admission, CABG or PCI in the past. In the context of forecasting, population transition probabilities that have been estimated for past years (eg 1992 to 1994) are extrapolated into future years for which forecasts are required (eg 1995 to 1999). These extrapolated probabilities are used within the model to simulate events among cohort members into the future (and taking into account the ageing of the cohort). At the end of the first cycle people could have moved to a different disease history state or moved into one of two possible death (absorbing) states, namely (5) death from CHD or (6) death from causes other than CHD. Within a cycle, our model allows for a CHD admission without a CARP, up to two admissions with CABG or PCI in addition to possible death (Figure 1). A more detailed description of the model and the estimation of population transition probabilities have been provided elsewhere [13].

For extrapolating transition probabilities two standard methods were used. These are (for each age, sex and history group) using the mean of the probabilities for the last three years (the mean method) and using linear extrapolation of transition probabilities based on the (weighted) annual changes over the last three years with twice as much weight assigned to the most recent annual change (the linear method). The mean extrapolation method is expected to perform well in situations when the (absolute) level of the probabilities remains fairly constant into the future and the linear extrapolation method is expected to perform well in situations when the probabilities are changing and are expected to continue to change at approximately the same rate. Modified extrapolation methods are obtained by modifying selected key probabilities of CARP events and adopting the standard extrapolation probabilities for the rest of the model probabilities.

The forecast number of events is based on the average of 100 replicate simulations of the entire cohort [13]. The accuracy of the total forecast number of CABGs and PCIs is evaluated using the percent over- or under- compared to the total actual number. The accuracy of forecasts across age and sex groups is evaluated by calculating a goodness-of-fit (GOF) observed-predicted chi-squared statistic. The smaller the GOF statistic the better are the model forecasts. We used SAS version 9.0 [14] for calculating the simulation inputs and TreeAge Data Pro 2005 for Markov simulation modelling [15].

No ethical approval was required for the project as the data for this project were already provided to the researchers by the Data Linkage Unit of the WA Department of Health.

## Results

Table 1 shows the number and history distribution of the WA 1995 population cohort. Overall 93.1% of men and 96.1% of women were in the 'no history of CHD' state but this varied from 99.2% in 35–39 year old men to 76.2% in 75–79 year old men and from 99.6% in 35–39 year women to 84.6% in 75–79 year old women.

**Table 1:** Age and sex distribution of the population of Western Australia for 1995

Sex	Age group	Hist no CHD	Hist CHD	Hist CABG	Hist PCI	Total Population
Male	35–39	70144	461	28	52	70685
Male	40–44	66197	818	100	142	67257
Male	45–49	61080	1290	294	314	62978
Male	50–54	44866	1689	506	445	47506
Male	55–59	34500	2048	779	563	37890
Male	60–64	27557	2522	1092	585	31756
Male	65–69	23852	2930	1309	576	28667
Male	70–74	17646	3097	1184	398	22325
Male	75–79	10297	2345	667	206	13515
						
Female	35–39	70024	301	6	4	70335
Female	40–44	66195	472	16	19	66702
Female	45–49	58333	632	29	46	59040
Female	50–54	42960	937	60	84	44041
Female	55–59	34614	1265	130	119	36128
Female	60–64	29418	1592	204	169	31383
Female	65–69	26848	2073	388	261	29570
Female	70–74	22407	2610	431	250	25698
Female	75–79	15694	2380	326	156	18556

Tables 2 and 3 show the actual and forecast number of CABGs and PCIs respectively for the standard models. The results show that the mean method overestimates the total actual number of CABGs by 20.5 percent and the linear method by 15.6 percent. The GOF values are reasonably close for the two methods. The forecast numbers of PCIs from the mean method under-estimates the actual number of PCIs by 15.3 percent and the linear method over-estimates by 17.7 percent. The GOF value is considerably larger for the mean method.

**Table 2:** Comparison between actual numbers of CABGs and forecast numbers (1995–1999) based on the standard Mean and Linear extrapolation methods as well as selected modifications of these methods

Sex	Age Group	Actual	Forecast under mean method	Forecast under modified mean method (Model 1)	Forecast under further modified mean method (Model 2)	Forecast under final modified mean method (Model 5)	Forecast under linear method	Forecast under modified linear method (Model 3)	Forecast under further modified linear method (Model 4)
Male	35–39	90	113	115	110	98	127	124	122
Male	40–44	206	271	278	265	239	271	266	262
Male	45–49	394	535	533	528	470	512	512	495
Male	50–54	601	733	730	719	637	697	696	678
Male	55–59	759	892	892	883	778	862	862	844
Male	60–64	829	1035	1028	1025	896	1009	1013	1007
Male	65–69	950	1075	1066	1065	930	1088	1078	1080
Male	70–74	678	771	773	769	667	810	808	810
Male	75–79	245	309	306	307	264	361	359	360
Male	All	4752	5734	5721	5671	4979	5737	5718	5658
									
Female	35–39	22	16	18	15	15	14	15	15
Female	40–44	40	38	41	38	36	37	40	38
Female	45–49	49	82	85	72	76	70	70	69
Female	50–54	108	137	143	125	128	126	127	123
Female	55–59	152	216	224	213	200	200	199	197
Female	60–64	262	305	324	302	281	242	243	239
Female	65–69	319	392	417	387	362	283	284	276
Female	70–74	302	341	363	336	313	264	267	263
Female	75–79	151	161	169	161	145	145	144	142
Female	All	1405	1688	1784	1649	1556	1381	1389	1362
									
Total		6157	7422	7505	7320	6535	7118	7107	6744
									
% change from actual			20.5	21.9	18.9	6.1	15.6	15.4	9.5
									
GOF			246.52	272.87	212.55	61.96	224.65	215.30	200.18

**Table 3:** Comparison between actual numbers of PCIs and forecast numbers (1995–1999) based on the standard Mean and Linear extrapolation methods as well as selected modifications of these

Sex	Age Group	Actual PCI	Forecast under mean method	Forecast under modified mean method (Model 1)	Forecast under further modified mean method (Model 2)	Forecast under final modified mean method (Model 5)	Forecast under linear method	Forecast under modified linear method (Model 3)	Forecast under further modified linear method (Model 4)
Male	35–39	233	204	210	190	236	251	243	241
Male	40–44	412	423	424	401	492	554	527	526
Male	45–49	730	697	696	659	817	934	889	893
Male	50–54	788	800	807	754	917	1032	1008	1014
Male	55–59	909	819	824	779	950	1084	1013	1014
Male	60–64	888	762	767	717	880	1069	994	994
Male	65–69	878	662	665	632	769	1064	988	991
Male	70–74	716	414	417	393	477	745	694	698
Male	75–79	260	153	153	146	177	261	259	262
Male	All	5814	4934	4963	4671	5715	6994	6615	6633
									
Female	35–39	24	19	21	20	23	26	26	25
Female	40–44	81	61	63	64	69	58	56	56
Female	45–49	119	134	136	138	148	135	129	128
Female	50–54	189	178	185	213	201	215	199	199
Female	55–59	250	221	230	222	249	285	262	260
Female	60–64	338	279	289	281	315	341	324	319
Female	65–69	381	326	339	329	364	436	411	414
Female	70–74	348	274	284	258	293	397	377	375
Female	75–79	197	129	136	122	134	219	205	205
Female	All	1927	1621	1683	1647	1796	2112	1989	1981
									
Total		7741	6555	6646	6318	7511	9106	8604	8614
									
% change from actual			-15.3	-14.1	-18.4	-3.0	17.6	11.1	11.3
									
GOF			494.11	455.09	643.38	265.37	265.03	155.37	159.38

**Figure 2:**
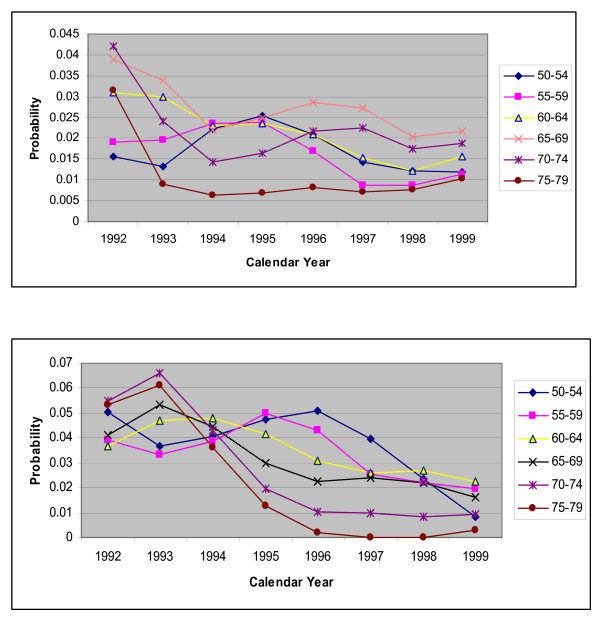
**a.** Plot for risk of CABG within same year following a PCI, for people with no history of CHD by calendar year, for males with different age groups. **b.** Plot for risk of CABG within same year following a PCI, for people with no history of CHD by calendar year, for females with different age groups

**Figure 3:**
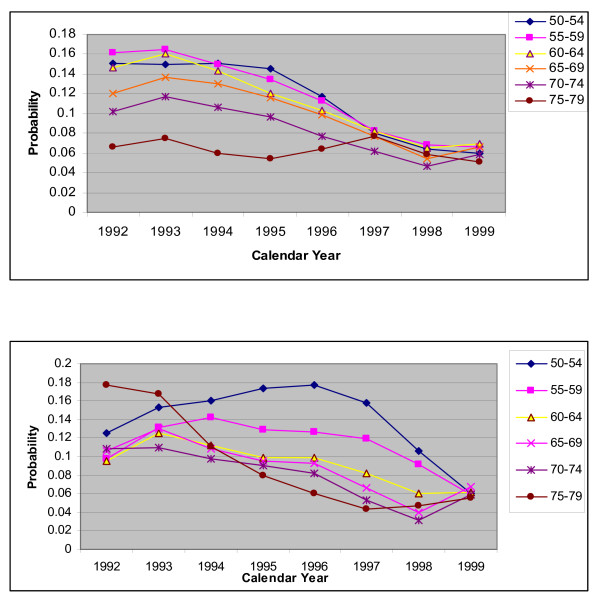
**a.** Plot for risk of a repeat PCI within same year, for people with no history of CHD by calendar years, for males with different age groups. **b.** Plot for risk of a repeat PCI within same year, for people with no history of CHD by calendar years, for females with different age groups

The poor performance of these standard methods for forecasting the requirements of CARPs over 1995–99 may be due to the fact that the effectiveness of PCIs increased after 1994 when stents were first introduced in WA and as a result the probability of requiring another CARP within the same year was reduced. Within the model these are represented by risk of a CABG or another PCI in the same year following a PCI procedure. For both males and females, the trends in these probabilities for the largest history group 'no history of CHD' are shown in Figures 2a, 2b, 3a and 3b. It is clear that the trend over the period 1992 to 1994 did not continue into 1995–1999 and hence the standard extrapolation methods would not be accurate for these probabilities.

**Figure 4:**
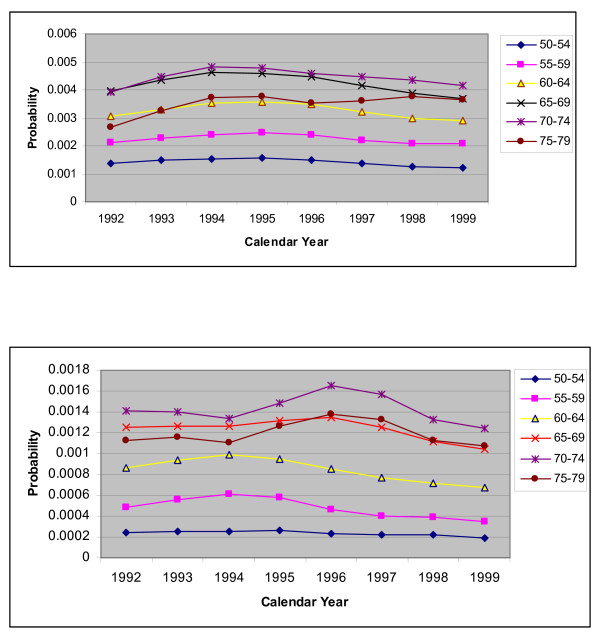
**a.** Plot for risk of a CABG within a year, for people with no history of CHD by calendar year, for males with different age groups. **b.** Plot for risk of a CABG within a year, for people with no history of CHD by calendar year, for females with different age groups

**Figure 5:**
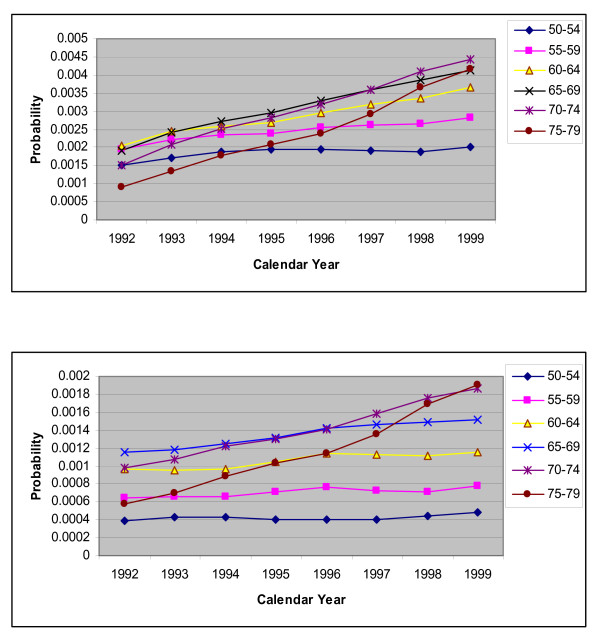
**a.** Plot for risk of a PCI, for people with no history of CHD by calendar year, for males with different age groups. **b.** Plot for risk of a PCI, for people with no history of CHD by calendar year, for females with different age groups

Another explanation for the poor performance of the standard methods was that there was an increasing trend in PCI rates during the early 1990s as the number of facilities performing PCIs increased and this continued until about 1995 [6]. This, together with the increasing effectiveness of PCIs meant that many more PCIs but fewer CABGs were being performed. Within the model these are captured by risk of a CABG and risk of a PCI. For both sexes, the trends in these risks for the 'no CHD history' group are shown in Figures 4a, 4b, 5a and 5b. It is clear that the risk of a CABG and that of a PCI gradually declined and increased respectively after 1994 for most age groups 50–54 and above (the trends for younger age groups are not shown as these risks are low), for both sexes.

In order to incorporate modifications to the standard extrapolation methods relating to the increased effectiveness of PCI procedures (ie the decreased risk of requiring a second CARP within the same year) we have examined the clinical trial evidence for the improved effectiveness of PCIs with stents. The results of a meta-analysis of 29 RCTs by Brophy et al. [16] indicate that risk of a repeat PCI would have declined by 41 percent in Western Australia from 1995 due to the introduction of stents. The observed risks for the Western Australian population showed that the risk of a CABG after a PCI also declined by 41 percent and thus we also reduce this risk accordingly.

To quantify the changed trends for PCI and CABG rates we use evidence from a population based observational study in Western Australia which showed that for males risk of a PCI increased by about 25 percent and risk of a CABG declined by about 15 percent when stents were introduced in WA in 1995 [5]. For females, there were similar shifts but the magnitude was about half that in males.

To accommodate these changed trends we have modified the standard extrapolated probabilities for these events by the amounts indicated by the evidence. Forecasts based on the following modified models have been obtained and the results are shown in Tables 2 and 3.

**Model 1**: the annual risk of a repeat PCI declines by 41 percent while the other risks are mean extrapolated,

**Model 2**: the annual risk of a repeat PCI and risk of a CABG given PCI decline by 41 percent while the other risks are mean extrapolated,

**Model 3**: the annual risk of a repeat PCI declines by 41 percent while the other risks are linearly extrapolated,

**Model 4**: the annual risk of a repeat PCI and risk of a CABG given PCI decline by 41 percent while the other risks are linearly extrapolated, and

**Model 5**: the annual risk of a repeat PCI and risk of a CABG given PCI decline by 41 percent and risk of a PCI increases by 25 percent for males or 12.5 percent for females with a corresponding decline in risk of a CABG by 15 percent for males or 7.5 percent for females while the other risks are mean extrapolated.

The results show that the forecast numbers of PCIs under Model 1 become closer to the respective actual numbers compared to standard mean extrapolation although the discrepancies are still generally quite large. Also, this modification does not improve the forecasts for CABGs. The forecasts for numbers of CABGs and PCIs under Models 2, 3 and 4 improve only marginally. There is over-estimation in the actual number of CABGs by 21.9, 18.9, 15.4 and 9.5 percents respectively under Models 1, 2, 3 and 4. There is under-estimation in the actual number of PCIs by 14.1 and 18.4 percents respectively under Models 1 and 2, and over-estimation by 11.1 and 11.3 percents under Models 3 and 4.

As the forecasts under Model 3 which use linear extrapolations seriously overestimated the numbers of PCIs the linear method was not further considered. Model 5, which also incorporates modifications to risk of a PCI and risk of a CABG has considerably better forecasts. There is now only 6.1 percent over-estimation of number of CABGs under Model 5 while for PCIs there is an under-estimation by only 3 percent.

## Discussion

In this paper we have examined forecasts for 1995–1999 based on extrapolation of trends from 1992–94. This was done because actual event numbers were known for these years and to illustrate the need to modify the standard methods in a period like this when there were rapid changes in CARP surgical technology and practice. However, the limitations of the retrospective nature of this study should be acknowledged. We believe that the modifications could have been anticipated in 1995 when knowledge of increased PCI effectiveness was becoming available and the changing preference for PCI over CABG was common knowledge among cardiologists and cardio-thoracic surgeons. However, quantifying the impacts of these expected changes in 1995 would have been more difficult than in our illustration which used evidence published after 1995.

Our CHD/CARP model does not directly incorporate the effects of risk factors such as blood pressure, cholesterol, obesity and smoking on event risks. Whilst cohort-based models that incorporate risk factors have been proposed (eg, Sesso et al. [17]) it is not practical to incorporate risk factors directly into our model because it has 96 different risks (for each age and sex group) and thus 96 risk factor models would be required. Further, as our model simulates individual pathways, such an approach would also require risk factor data on the entire population of Western Australia. However, our model can still indirectly incorporate changes in trends in risk factors by appropriate modification of model probabilities that relate to risk of certain CHD events (eg for people currently with no history of CHD).

Using the approach of modifying selected and relevant model event probabilities, our model is flexible enough to explore the effect on the future requirements of CARPs of a variety of changing circumstances. In addition to advances in surgical procedures and changing preference for surgical procedures as we have illustrated, other scenarios such as changes in the effectiveness of, and access to, medical treatments may be investigated. For example, randomized controlled trials have shown that there were significant reductions in requirements for CARPs among higher risk individuals who were given antiplatelet therapy versus controls [18]. An example of a possible change in health policy related to treating CHD patients could be the availability of low cost statins [19]. Since the cost of the Australian Pharmaceutical Benefit Scheme which subsidizes the cost of statin drugs has been increasing rapidly, the government may change its current policy and only provide subsidy to people at higher risk of CHD. The effect of these changes relating to medical treatments could be investigated using our CHD/CARP model through modifications to selected and relevant model probabilities.

The CHD/CARP model can also be extended and used to determine the cost of future requirements for CARPs and to explore the most cost-effective strategies for treatment of CHD. To achieve this, indicative cost estimates are needed for each of the alternative CARP treatments for each year. For more description of cost effectiveness analysis one can refer to some studies which have evaluated economic impact of drug-eluting stents [20,21], and cost effectiveness of CHD prevention through CHD risk reduction strategies [22].

## Conclusion

It is clear from the model simulation results that forecasting events in an era of rapid changes is fraught with difficulties and standard extrapolation methods are not likely to perform well in such situations. However, there is often knowledge and sometimes quantitative evidence of the expected impacts of changes in procedure effectiveness and practice and these may be used to modify the standard forecasting methods to achieve better forecasts as we have illustrated. The simulation results implied that modifications that related to the changing relative use of a PCI to a CABG were more important for correctly projecting the numbers of CARPs than modifications that related only to the increased effectiveness of PCI procedures.

## Competing interests

The authors declare that they have no competing interests.

## Authors' contributions

HRM did all aspects of this research work as part of his doctoral dissertation including model design, development, implementation, and analysis and also wrote the draft of this paper. MK supervised the research project giving valuable inputs in the model design and concept and finally helped draft the paper. MH was involved as a co-supervisor.

## Pre-publication history

The pre-publication history for this paper can be accessed here:


